# Alginate: Enhancement Strategies for Advanced Applications

**DOI:** 10.3390/ijms23094486

**Published:** 2022-04-19

**Authors:** Alejandro Hurtado, Alaa A. A. Aljabali, Vijay Mishra, Murtaza M. Tambuwala, Ángel Serrano-Aroca

**Affiliations:** 1 Biomaterials and Bioengineering Laboratory, Centro de Investigación Traslacional San Alberto Magno, Universidad Católica de Valencia San Vicente Mártir, c/Guillem de Castro 94, 46001 Valencia, Spain; alejandro.hurtado@mail.ucv.es; 2 Department of Pharmaceutics and Pharmaceutical Technology, Faculty of Pharmacy, Yarmouk University, Irbid 21163, Jordan; alaaj@yu.edu.jo; 3 School of Pharmaceutical Sciences, Lovely Professional University, Phagwara 144411, Punjab, India; vijaymishra2@gmail.com; 4 School of Pharmacy and Pharmaceutical Science, Ulster University, Coleraine BT52 1SA, Northern Ireland, UK; m.tambuwala@ulster.ac.uk

**Keywords:** alginate, biopolymer, hydrogel, enhanced properties, composites

## Abstract

Alginate is an excellent biodegradable and renewable material that is already used for a broad range of industrial applications, including advanced fields, such as biomedicine and bioengineering, due to its excellent biodegradable and biocompatible properties. This biopolymer can be produced from brown algae or a microorganism culture. This review presents the principles, chemical structures, gelation properties, chemical interactions, production, sterilization, purification, types, and alginate-based hydrogels developed so far. We present all of the advanced strategies used to remarkably enhance this biopolymer’s physicochemical and biological characteristics in various forms, such as injectable gels, fibers, films, hydrogels, and scaffolds. Thus, we present here all of the material engineering enhancement approaches achieved so far in this biopolymer in terms of mechanical reinforcement, thermal and electrical performance, wettability, water sorption and diffusion, antimicrobial activity, in vivo and in vitro biological behavior, including toxicity, cell adhesion, proliferation, and differentiation, immunological response, biodegradation, porosity, and its use as scaffolds for tissue engineering applications. These improvements to overcome the drawbacks of the alginate biopolymer could exponentially increase the significant number of alginate applications that go from the paper industry to the bioprinting of organs.

## 1. Introduction

Alginate has established itself as an excellent biodegradable, renewable, and biocompatible material with non-immunogenicity and easy gelation capacity [1,2]. This biopolymer can be produced from brown algae or microorganism culture [3,4]. Alginate is a U.S. FDA-approved product currently used in several medical applications, such as thickening stabilizing agents for dental impression materials and wound healing treatments in gel form [5]. Alginate currently possesses an immense number of industrial applications in a wide range of fields, such as biotechnology, bioengineering, biomedicine, and clinical applications, the pharmaceutical industry, chemical, textile, packaging and construction industry, food and drinks, aquaculture, dentistry, paper industry, arts and crafts, and the leisure industry (Table 1).

Biotechnology, bioengineering, biomedicine, and clinical areas include dressings for wound healing [6,7], heavy metal chelators [8,9], tissue engineering [10,11,12,13], control drug delivery [14,15], 3D bio-printing [16,17], prosthesis, dental molds and impression materials [18,19], and cell immobilization [2,22]. In drug delivery, alginate hydrogels can efficiently encapsulate compounds such as insulin [53] or diverse enzymes [54] to be released because they possess adequate porosity for these purposes. Alginate can be used as a carrier of proteins to protect them from degradation and minimize denaturation [55]. There is an increasing use of alginate hydrogels in the human body, resulting in no adverse effects [56]; these gels have great potential in tissue regeneration, such as bone, muscle, nerve, pancreatic, or hepatic tissue, in combination with cell transplantation and/or delivery of growth factors [57,58,59]. The pharmaceutical industry includes food supplements [23], treatment for gastric reflux [24,25], and cancer therapy [26,27]. Alginate can be combined with drugs (e.g., ketoprofen) in mixtures to exhibit anti-inflammatory effects after oral administration for colon diseases [60].

Alginate has also shown great potential in the chemical, textile, packaging, and construction industry, including cosmetics [28,29], textile inks [30,31], detergents [30,31], adhesives [33,34], welding [35,36,37] and building insulation [38]. Alginate has shown great potential in developing biodegradable packaging using this biopolymer in the form of films [39]. Other fields of application of this biopolymer include food and drinks, such as ice creams and beer, aquaculture, arts and crafts, and the paper and leisure industry.

However, even though there are currently many industrial applications, alginate hydrogels possess very weak mechanical properties, especially when they are hydrated in water. These materials have very low electrical and thermal conductivity, non-antibacterial activity, and very poor cell binding activity, which is essential for different advanced applications, such as tissue engineering. Therefore, if these poor properties are enhanced, the application fields of this biopolymer can be increased even more in biomedicine and other industrial fields where the mechanical performance, electrical and/or thermal behavior, water sorption and diffusion, in vitro and in vivo biological functionality, antimicrobial activity, and porosity are essential. In this sense, much research on alginate combined with other materials has been focused on enhancing its chemical, physical, and biological properties. Therefore, alginate-based materials have been fabricated with a broad range of different hybrid polymeric structures based on interpenetrating polymeric networks (IPN) [61,62,63], semi-interpenetrated networks (semi-IPN) [64,65], or combined with other materials, such as fibers, nanofibers, metallic, ceramic, and polymer nanoparticles, and carbon nanomaterials, including graphene (Gr) and its derivatives [4,66,67,68,69,70,71,72,73,74]. Furthermore, porous alginate-based supports (scaffolds) for regenerative medicine are being developed with an improved structure composed of pores with different shapes, interconnection, and porosity degrees, depending on the application, and generated by advanced methods, including bioprinting [17,75,76,77]. Several reviews on alginate-based materials have recently been published for environmental [78], food and packaging [46,79], heavy metals and radionuclides adsorption [80], and biomedical applications [81], in particular, in wound dressing [82], drug delivery [83], and tissue engineering [84,85,86]. However, in this review, we present a profound journey to learn the principles of the alginate biopolymer, its chemical structure, gelation properties and interactions, production, and purification, and the types of alginate-based hydrogels developed so far. A recompilation of advanced procedures to remarkably enhance the physicochemical characteristics and biological activities of the alginate materials in various forms, such as injectable gels, fibers, microcapsules, films, hydrogels, etc., and scaffolds are shown here for the first time in the literature. Thus, we present here all the enhancement strategies of this biopolymer achieved so far in terms of mechanical reinforcement, thermal and electrical performance, wettability, water sorption and diffusion, antimicrobial capacity, in vivo and in vitro biological behavior, including toxicity, cell adhesion, proliferation and differentiation, immunological response, biodegradation, porosity, and its use as a scaffold for tissue engineering applications. These enhancement strategies could exponentially increase the already immense number of alginate applications in a broad range of industries, from the paper industry to the bioprinting of organs.

## 2. Alginate: Chemical Structure, Gelation, Properties, Production, Types, and Purification

### 2.1. Chemical Composition

**Figure 1:**
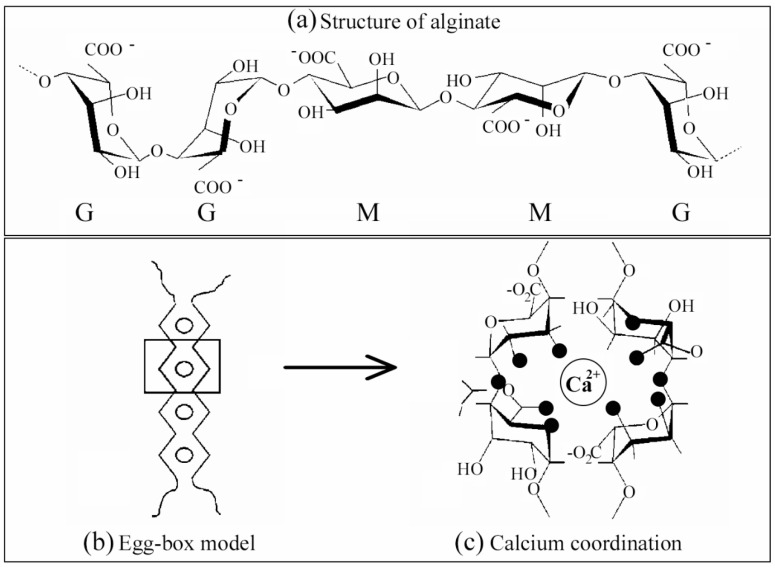
Chemical structure of alginate and alginate gelling: (**a**) β-(1→4)-D-mannuronic acid (M) and α-(1→4)-L-guluronic acid (G) blocks; (**b**) egg-box model; (**c**) calcium coordination described by the pair of guluronate chains in calcium alginate junction zones. Dark circles represent the oxygen atoms involved in the coordination of the calcium ion. Adapted with permission from reference [93]. Copyright 2007 American Chemical Society.

Sodium alginate (SA) is a compound that belongs to the group of polymeric sugars and is usually called alginic acid [87]. This biopolymer is a carbohydrate polymer [88] in the form of linear polysaccharides formed by a random sequence of two acid blocks, α-L-guluronic acid (G), and the β-D-mannuronic acid (M) presented in the form of blocks with mannuronic and guluronic (M/G ratio) at different proportions [89,90] and linked with an o-glycosidic bond (1-4) [91]. Alginates are composed of these two types of molecules distributed in different dispositions [92]. Figure 1a shows a short alginate chain’s chemical structure and the M and G blocks that make up the alginate structure. 

### 2.2. Alginate Gelation

Alginate polymer chains can generate gels that can be manufactured by acid precipitation or ionic reticulation, called ionotropic gelation [94]. Precipitation of acids to form alginate gels occurs as soon as the pH is reduced to parameters lower than the pKa or a dissociation coefficient of the biomaterial. Alginate has a negative charge over a fairly large pH spectrum [95]. The lowering of the pH rate impacts the alginate mixture in a couple of ways: (1) the precipitation of alginate generating those aggregates is caused when the pH is rapidly reduced; (2) the alginate gel is generated with a gradual and more stable reduction of pH [96]. Another factor is the consistency of the alginate achieved by molecular bonding of hydrogen and having the M-block residue factor in as they act appropriately in the mechanism of gel formation. However, gelation by ionic cross-linking to form ionic gels is caused when carboxylic group residues possessing a negative charge interact with divalent or trivalent cations or cationic materials [97,98]. The formation of alginate gels is mainly due to abundant GG blocks, which are responsible for the specific anionic bond, and the gelling properties of alginate hydrogels come from there [99]. It should be noted that the units of the MM block form a linear polymer, whereas those of the GG block form loop-shaped or rotational groups (see Figure 1a) [100]. The ability of SA to form gels when it is immersed in a solution containing divalent cations such as Ca^2+^ is the fundamental concept for using this biomaterial in different fields, including biological and technological fields [101]. Alginate has many carboxyl and hydroxyl groups in its polymeric structure, making alginate a suitable precursor, since it acts as a chelating agent [102]. Gelation by ionic crosslinking is generated by applying two external or internal techniques. These two vary in the divalent cations applied and in the gel formation in terms of kinetics. First, the external way is based on introducing the ions with an external base to generate the gels, when the alginate “sol” phase, typically in the droplet structure, penetrates the solution formed by water, the ions in the form of salt. The latter quickly reach the “sol” phase and are dispersed mainly on the surface, generating hydrogels of mixed form, where in the more central parts of the gel, there is less concentration of divalent ions. However, the inner gelling mechanism usually introduces ions with a quiescent mechanism into the “sol” phase. Thus, many examples of this technique include calcium, non-soluble minerals, such as phosphate, calcium oxalate, citrate, and carbonate. The established and controlled changes in the characteristics of the overall complex, such as variations in pH or the solubility capacity of ions, promote their release and generate the onset of crosslinking, generating simple, non-heterogeneous hydrogels with a correct dispersion of these ions [94]. However, the obtained beads are more sensitive to breakage [83]. Thus, alginate, in a medium with different divalent cations, such as calcium, zinc, or magnesium, among many others, forms the so-called “egg-box” structure (Figure 1b) [103].

Thus, hydrophilic alginate gels are generated by applying the characteristic cross-linking of SA using different types of divalent charged cations, such as Ca^2+^, Zn^2+^, Mg^2+^, etc. [104]. Alginate hydrogels can swell with a three-dimensional (3D) shape without being solved when they are in an aqueous medium. The divalent cations induce intercommunication and gel bonding zones [93]. In this cross-linking process, two G-block structures are “linked” to each other by the union of a divalent cation (Figure 1c) [103]. Random GM blocks also contribute to the bonding zone and, thus, form this characteristic structure [105]. The so-called egg structure has also been reported to be formed by chains of four consecutive G-blocks [106].

On the other hand, gelation kinetics and features may vary depending directly on temperature [107], and one of the complications is that the ions must be known since the properties are related to characteristics such as valence or atomic radius [94]. Ion binding in isolation is a requirement for the hydrogel, so the constitution and order of monomers have great value in the characteristics of the formed calcium alginate hydrogels [108]. The alginate structure can be transformed into macroporous epichlorohydrin cross-linked alginate beads, acquiring the property of absorbing proteins [109].

Alginate gelation can occur by other methods, such as photo-crosslinking, thermal gelation, or a synergistic mixture of these methods [110]. This biomaterial allows cross-linking with compounds, such as glutaraldehyde or other mixtures [111]. These cross-linking approaches lack a level of safety comparable to ion cross-linking, especially for use in biomedical techniques, as they may contain some toxicity that precludes their use. The process may be approached with photo-crosslinking, even with the proper chemical starters [42]. For example, the use of argon ions for crosslinking with alginate in combination with methacrylate produces clean and flexible hydrogels, used to seal a corneal perforation in vivo, demonstrating yet another use in surgical techniques without the need for sutures [112]. Alternative methods of photo-crosslinking usually involve the use of a light sensitizer or the release of acid, which can be hazardous to the organism [58].

Problems can be encountered to produce homogeneous alginate-based materials when using external cation crosslinking solutions. For example, to produce homogeneous films of calcium alginate, it is necessary to produce films of sodium alginate by solvent casting, followed by immersion in a calcium chloride aqueous crosslinking solution [67,69,71]. The crosslinking solutions must be prepared with the suitable concentrations of divalent cations and immersion time must be controlled in order to tailor the required mechanical and water sorption properties.

### 2.3. Alginate Properties

The physicochemical properties of alginate may be modified because they depend on the conformation of the biopolymer [113]. The mannuronic and guluronic building blocks that make up the alginate structure and the conformation and partitioning in the alginate structure must be considered to vary the alginate characteristics [39]. There are up to 200 different types of alginate with different molecular mass and/or chemical compositions of the associated elements [114]. Alginates show different percentages of glycosidic monomers that make up the alginate structure [115]. Therefore, its applications depend on its properties and are directly proportional to its structure type. On the other hand, alginates are non-toxic, biocompatible, and biodegradable materials [116]. The ability to degrade alginate is impossible in mammals, mainly because the enzyme necessary for the process is not present [117]. However, degradation does occur in alginate hydrogels cross-linked with ions as there is an outflow of ions into the environment with exchange reactions. The degradation rate of alginates can be controlled and may be very slow, preventing degradation in the short-term [118]. Other important physicochemical properties of alginate involve selective bonding of divalent cations, the basis of hydrogel formation [119]. The pH is essential for the solubility of alginates [119]. Thus, a very low pH causes the precipitation of alginate chains [120]. Any variation in ionic strength is likely to affect solubility and viscosity; dispersion and solubility will vary with different ionic strengths [121]. Thus, alginate will have another state depending on pH: alginate becomes a gel at neutral pH, alginate swells at pH 3, and alginate becomes more viscous at pH 8 [122]. The principles of the properties of alginate gelling are based on this simple characteristic [123]. The selectivity of alginate blocks is very relevant as G-blocks are more efficiently bound to divalent ions than M-blocks [124]. In addition, G-blocks have a significant value because they have an overall characteristic that they can be presented as regulators or modulators of gelation when dissolved with gelling alginates [125]. The selectivity of this polysaccharide for positive ions with double valence is precise to the number of ions in the natural biomaterial hydrogel [126].

The control mechanism for the union of alginates with ions becomes a procedure of great importance for its final structure [127]. The mediated introduction of binding cations is made possible by two mechanisms for preparing an alginate natural biomaterial hydrogel: the dissemination and the inner fixation methods [119]. The dissemination process differs because it allows the introduction of a crosslinking cation from a large external reservoir into an alginate solution [128]. The diffusion setting is characterized by a much faster gel rate and is applied for immobilization purposes, where each of the polysaccharide dissolution droplets makes a single drop of gel with an active agent included within it [127]. The internal method differs from diffusion by the mediated secretion of the cross-linking ion already inside the hydrogel [129].

Properties of the material, such as stability and ionically cross-linked gels, are essential for biological use [130]. Alginate is sensitive to depolymerization because it is composed of simple chain polymeric material [130]. Thus, the stability of glycosidic bonds is variable but can be broken by techniques that apply pH variation or oxidation with free radicals [131]. Hydrolysis of the gel occurs when complex stability is reduced to a pH below 5 because there is a higher concentration of protons; instead, decomposition is generated at a pH above 5.

Autoclaving should not be used to sterilize SA. γ-radiation leads to irreversible alginate damage with hydroxyl free radical (OH*) formation [132]. Since this polymer can be dissolved in water at ambient temperature, sterile filtration is recommended. Crosslinked alginate in the form of films can be sterilized by cleaning with absolute ethanol and subsequent UV irradiation for 1 h per side [68,71,104,133,134,135].

### 2.4. Alginate Production

Currently, alginate biosynthesis can arise from brown algae, such as *Laminaria Hyperborea*, or bacteria, such as *Pseudomonas aeruginosa* [136].

#### 2.4.1. Alginate Produced by Brown Algae

**Figure 2:**
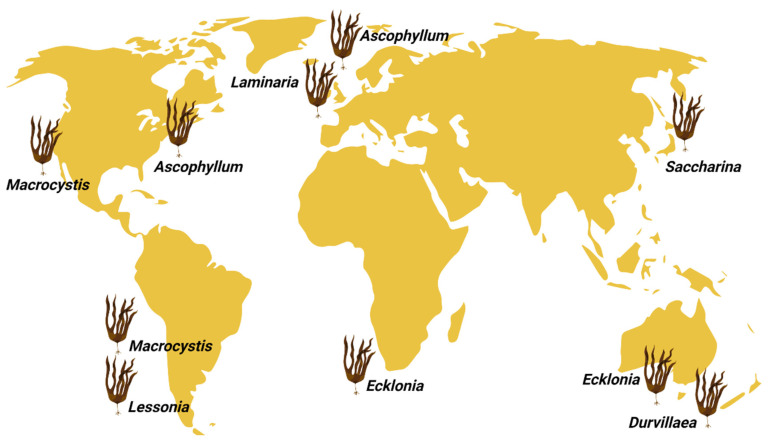
Worldwide geographical areas where some types of algae are used for alginate production. Created with BioRender.

The generation of brown algae began in California in 1929; soon after, in 1939, it was developed in some European countries and Japan [137]. At present, there is a high regional imbalance in seaweed production [138]. Thus, 35.8 million tons of global seaweed production is contributed to by 49 countries/territories and 97% of the world production comes from Asia. Thus, the major producer countries per area are China, Indonesia, Republic of Korea, and Philippines in Asia. In Americas, Chile is the major producer at the same level of Japan. In Europe, Norway and France are the major producers but with production rates three and eight times lower than that of Japan, respectively. Alginates are derived from brown seaweed and are produced mainly in the USA, Norway, China, Canada, France, and Japan [139]. Among the advantages of obtaining alginate from brown algae is that most alginophytes have large quantities of the product and generate large areas on the rocky shores with little depth [140,141]. Figure 2 shows the areas where some algae used for alginate production are found throughout the world [142].

Alginate is found in algae, mainly in the cell wall and especially in species such as brown algae [143]. Alginate extraction from brown algae is carried out based on various mechanisms, such as applying various centrifuges, variations in pH, and allowing precipitation [99]. The yield of alginate extracted from brown algae is about 44.32% on average [144]. More than 200 types of alginate are commercially available, and most of them are extracted from wild brown seaweed [58]. Among the species of brown algae from which alginate is extracted, *Macrocystis pyrifera* is one of the most essential [145]. Among the species that produce alginate, from the industrial point of view, the most relevant are *Ascophyllum nodosum*, *Macrocystis pyrifera,* and *Laminaria hyperborea* [142]. A novel method is used to cultivate brown algae in the laboratory using free-living gametophytes, as they have advantages in terms of genetic selection capacity, clone generation, and production of large quantities [142,146]. The relative molecular mass of alginate ranges from 32 to 400 kg/mol [147], and the leading causes of variability are the algae’s species and age [148]. Table 2 shows the main types of alginate extracted from different brown algae.

Other alginate sources not included in the table are *Ecklonia maxima* and *Lessonia nigrescens* [160].

#### 2.4.2. Alginate Produced from Bacterial Culture

Alginate is a bio-polysaccharide that was first isolated from marine macroalgae, but later, this sugar was observed in differentiated bacteria [161]. Alginate is an essential component of the biofilm of bacteria that produce pulmonary fibrosis disease [162]. Of the bacterial types, the most relevant for alginate production are the species *Pseudomonas aeruginosa* [163] and *Azotobacter vinelandii* [164]; they are capable of producing large amounts of this biopolymer as an excretion polysaccharide in the bacterial biofilm [163]. In the first bacterium, alginate has a relevant role in the biofilm structure, and *A. vinelandii* uses alginates that possess high G-block and low M-block concentrations to be more persistent in cysts [165]. The PMI-GMP isomerase enzyme is fully involved in different early and late alginate production processes in these bacteria [166]. Multiple genes are found in the alginate generation pathway necessary for its production (Table 3), some of which are responsible for the proper functioning of alginate polymerase [163].

Some genes are not directly related to alginate synthesis but produce alternative proteins [180]. Alginate acts as an inducer of increased alginate production in a positive feedback reaction at the gene promoter of the biosynthesis pathway [170]. These genes could be used for molecular modifications in genetic engineering [181]. Alginate matrix is governed by an enzyme called mannuronan-C-5 epimerase that interconnects stereo-specific epimers [182]. Thus, alginate can be chemically modified by acetylation and epimerization, located in the time interval in which they are transferred to the periplasmatic space [183]. Genetic engineering and protein engineering can be used to produce bacterial alginates by modeling bacterial genetics [165]. Finally, it should be noted that only alginates produced from bacterial cultures have a greater capacity to interact with water molecules because the glycosidic product is found with acetylation at carbon positions 2 and 3 [164]. The main alginate types produced from different bacterial cultures, M/G ratios, and bacteria features from bacteria are shown in Table 4.

As mentioned above, there are multiple genes involved in synthesizing these genes; up to 24 stand out in the bacterium *P. aeruginosa* [167]. Most of these genes are found on the bacterial chromosome, and so far, there are no studies that show that it is located in plasmids [180]. There is a tremendous advantage in alginates produced by bacteria over alginates produced by algae because the quality generated is much better, and their characteristics and activities have been studied in detail [97,189]. However, alginate produced from bacterial culture presents several disadvantages concerning alginate extracted from brown algae. Thus, in the first place, the production of bacterial alginate is much more expensive than that produced from brown algae [190]. Furthermore, extraction is interrupted by the enzyme alginate lyase, and, in addition, the mucoid strains of *P. aeruginosa* often stop producing alginate when grown in the laboratory [136]. Furthermore, the alginate produced by bacteria has the existence of the enzyme epimerase that hinders the procedure [136,191].

### 2.5. Alginate Purification Methods

After extraction, the alginate contains residues, such as heavy metals, protein compounds, toxins, and polyphenols [97] that compromise the biocompatibility of this biopolymer [147,165]. Therefore, accurate extraction techniques must be performed, eliminating upstream and unnecessary compounds, and purification must be performed to eliminate downstream compounds, avoiding an immunogenic response in biomedical applications [83]. Alginate used in medicine and introduced into the body without purification leads to cell overgrowth around the capsules of this biopolymer, so purification techniques must be used to reduce contaminants such as immunogenic proteins [192,193]. Unpurified alginate, raw alginate, in a sphere for directed microencapsulation introduced into living organisms, is also known to produce characteristic pathogen-associated molecular patterns (PAMPs) and damage-associated molecular patterns (DAMPS) that stimulate the immune response [53]. The viscosity must also be considered within alginate purification, as this parameter is affected after purification [194]. Alginate can be purified by filtration, extraction, and precipitation [195]. A novel alginate purification method is the chemical purification procedure applied to alginates with variations in the proportions of M-monomers and G-monomers [196]. It improves stability and reduces diffusion, permeability, and increases the practical defense of quiescent cells against the body’s response to the immune process in such a case [197]. Correct elimination of downstream compounds decreases immunogenic factors promoting the non-activation of the immune system [198]. Some of these impurities include endotoxins, certain proteins, and polyphenols [198]. Polyphenols can be dangerous for humans as reported by the World Health Organization, and can possibly accumulate in the body. Endotoxins and proteins have been associated with a reduced biocompatibility of the alginate. Thus, a considerable reduction of proteinic contaminants is obtained using size exclusion chromatography for alginate purification [199]. Another technique for alginate purification is the removal of mitogenic compounds by free-flowing electrophoresis [200]. The purification of alginate for clinical use has increased biocompatibility [201]. Many researchers prefer to buy ultrapure alginate directly from specialized companies, such as NovaMatrix-DuPont [202], to reduce work time. These companies offer well-characterized ultra-purified alginates with different molecular weighs and M/G rations in sterile conditions. Peptide-coupled alginates with enhanced cell adhesion properties can also be purchased.

## 3. Enhancement of Physicochemical Properties

A broad range of material engineering enhancement approaches have been achieved so far in the alginate biopolymer, in terms of mechanical reinforcement, thermal and electrical properties, wettability, and water sorption and diffusion.

### 3.1. Mechanical Reinforcement

The mechanical performance of alginate increases at higher concentrations [197] and higher G block contents with respect to the amount of M blocks [203]. Even though alginates possess excellent properties, such as biodegradability, biocompatibility, etc., their mechanical performance is relatively poor, especially in the hydrated state [204]. In this regard, many material engineering approaches have been developed, such as reinforcement combining alginate with other compounds, polymers, by nanofilling advanced materials such as nanofibers, carbon nanomaterials, nanometals, nanocellulose, or nanoclay, among others.

#### 3.1.1. Reinforcement with Other Polymers

Several multicomponent polymer structures using alginate have been developed, such as mixtures [205], graft copolymers [206,207], semi-interpenetrating polymer networks (semi-IPNs), such as alginate/chitosan [65], calcium alginate/polycaprolactone [208], or sodium alginate-g-poly(sodium acrylate) [64], and the full interpenetrated polymer network (IPN), such as alginate/poly(acrylic acid) [62,63] or sterculia gum/calcium alginate [61]. In addition, to achieve mechanical reinforcement, other enhancements of properties can be achieved by these multicomponent systems, such as heat-resistant capacity [209], cell adhesive properties [65], sensibility as drug delivery systems [95], etc. Different polymers such as cellulose acetate phthalate (CAP), polyphosphate (PP), sodium carboxymethylcellulose (CMC), cellulose sulphate (CS), and dextran sulfate (DS) have mixed with alginate to enhance its mechanical properties [210,211]. The microcapsules formed with alginate that are loaded with insulin were reinforced with another polymer, such as chitosan to increase insulin protection [211]. The mucoadhesive properties of chitosan have been combined with alginate [212]. Alginate can also be mixed with polymers of different chemical nature, such as those belonging to or derived from cellulose, acrylic polymers, pectins, and polyvinylpyrrolidone [213]. The mixture of polyethylene glycol acrylate with alginate has successfully promoted chemical stability for Langerhans microencapsulation of the islets for insulin release [214].

In recent reports, benzoyl peroxide has generated a polymerized matrix of itaconic acid with SA as an improvement [206]. Other investigations highlighted that using alginate with other compounds to generate a stable polymeric matrix has a significant value. Polymers such as poly(N-isopropylacrylamide) (PIPAAm) and poly (acrylic acid) (PAA) have been used to generate spheres by processes such as microwave-mediated secretion of indomethacin (IND) [207]. Hydrogels formed by semi-IPN have been developed using alginate and other common polysaccharides, hyaluronic acid [215] or chitosan [65]. Other semi-IPNs able to trap glucose isomerase consist of hydrogels that intertwine polyacrylamide and alginate chains [216]. A very recent study has reported the use of alginate and xanthan gum to produce a heart patch [217].

#### 3.1.2. Reinforcement with Fibers and Nanofibers

The mechanical properties of alginate-based hydrogels can be improved with the incorporation of fibers and nanofibers. Bonding with fibers and nanofibers promotes the improvement of properties, such as tensile strength, elongation, breakage, and compression resistance [218]. Sodium alginate-polyvinyl alcohol hydrogels have been reinforced with cellulose nanofibers [219]. These composites showed enhanced density, viscoelasticity, and mechanical strength: 79.5 kPa in compressive strength, 3.2 times higher than that of the neat hydrogel. Cellulose nanofibers and bio-extracts showed synergistic effects for fabricating high strength sodium alginate-based composite bio-sponges with antibacterial properties [220]. Cellulose fibers have been used to reinforce a bioadhesive formulation based on a combination of gelatin and alginate crosslinked with water-soluble carbodiimide [221]. These composite materials showed a dramatic increase in the viscosity and in the burst strength. Cellulose fibers and cellulose nanowhiskers isolated from mulberry pulp were used with alginate hydrogels to increase mechanical properties and tensile strength [222]. The bonding of alginate hydrogels with cellulose nanofibers (NFC) and microfibrillated cellulose (MFC) reinforced mechanical properties, tensile strength, and reduced water vapor permeability [223,224]. Nanocrystalline nanocellulose were used with alginate hydrogel films to significantly increase tensile strength, water vapor permeability, and molecular interactions [225]. Alginate hydrogels bonded with nanocellulose to provide excellent thermal stability and high water resistance have also been reported [226]. Improved mechanical performance of alginate hydrogels can be achieved with cellulose nanocrystal(s) (CNC) and cellulose nanocrystal oxide (OCNC) [73]. Hydrogel alginate spheres, together with magnetic nanocellulose, were used to improve drug delivery and release, mechanical reinforcement, and specific physicochemical characteristics of the final complex [227].

The mechanical properties of an alginate hydrogel were improved by reinforcement with ethanol-treated polycaprolactone nanofibers [228] and with aligned electrospun gelatin nanofibers [229]. Thus, the alginate/gelatin nanofiber hydrogel increased up to 541% in tensile modulus and 1690% in tensile strength, while keeping good transparency. Fiber reinforcement is also used in additive manufacturing. Thus, a 3D-printed fiber-reinforced hydrogel composite consisting of a combination of alginate/acrylamide gel precursor solution and an epoxy-based UV-curable adhesive can be fabricated [230]. Alginate was mixed with nanocellulose to allow 3D printing as a high-printability ink for fabric scaffolds [75,76] and 3D bioprinting with NFC [17,77].

#### 3.1.3. Reinforcement with Carbon Nanomaterials

**Figure 3:**
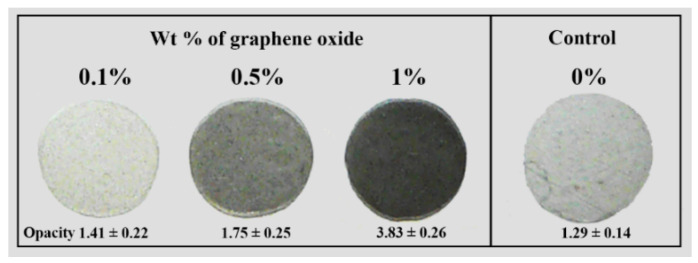
Film composite structures formed by calcium alginate and graphene oxide (GO). Increasing concentrations of GO in the range of 0–1% *w*/*w*. The opacity is presented as mean ± standard deviation in the base part of the samples. Reprinted with permission from Elsevier [71].

Calcium alginate films were synthesized with the incorporation of several amounts of carbon nanomaterials (CNMs), such as carbon nanofibers (CNFs) and graphene oxide (GO) [4,66,67,68,69]. These composite structures showed enhanced mechanical performance and much faster water diffusion through the carbon nanochannels formed in the alginate polymeric matrices. Thus, reinforced calcium alginate/GO composites have been developed (Figure 3) [71].

Compared with calcium alginate without any reinforcing agent, a nine-fold increase in the compression modulus of calcium alginate is seen when dry and up to a six-fold increase when hydrated for 1% *w*/*w* of GO. According to Figure 3, the opacity of alginate films with GO increases with the addition of GO to the composite. When little GO loading (0.1% *w*/*w*) is introduced into the alginate, the compressive modulus significantly increases. The addition of GO with an increase of calcium ions allows to generate alginate gels with a significant improvement in the mechanical reinforcement characteristic, which can be up to a multiple of four times the increase of this parameter [4]. A similar effect is presented when CNFs are used to reinforce calcium alginate [66]. In addition, the water absorption of the final composite increases with a very low amount of CNFs introduced into the alginate polymeric matrix. However, GO nanomaterial is more expensive than CNFs, so it is a factor that must be considered to produce these materials at a large scale.

**Figure 4:**
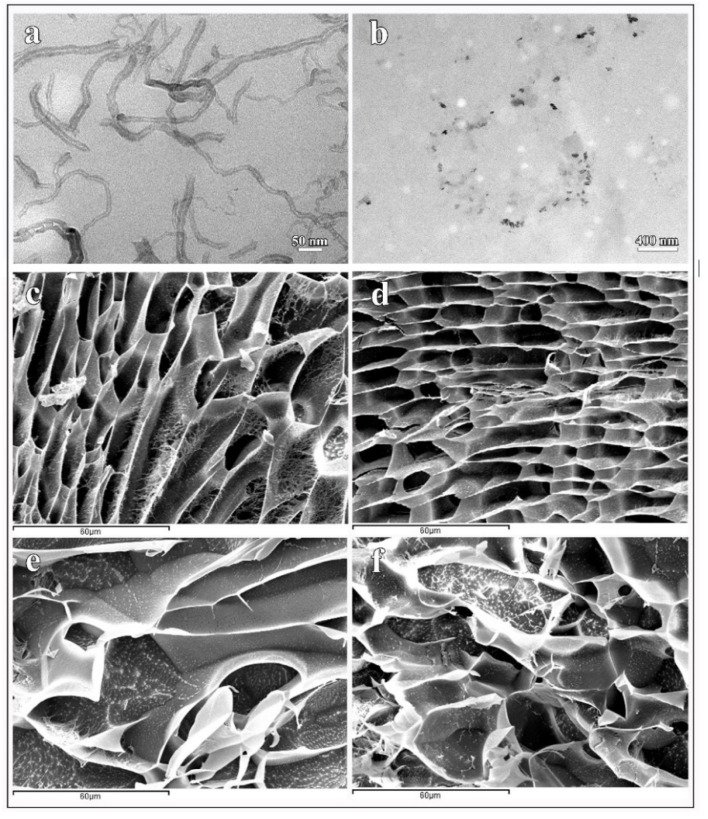
Transmission electron microscopy (TEM) captures of carbon nanofibers (CNFs) (**a**) and ultrathin sections of calcium-cross-linked alginate with CNFs at 1% *w/w* ratio (**b**); cryo-scanning electron microscopy (cryoSEM) of calcium-crosslinked alginate films, neat (**c**,**e**) and with CNFs (**d**,**f**), hydrated at 26 ± 0.5 °C and 37 ± 0.5 °C for 1 day, respectively. Reprinted with permission from Elsevier [68].

Carbon nanofibers are CBNs with a high degree of hydrophobicity [231]. Therefore, the composite structure generated by incorporating CNFs into the hydrophilic alginate polymer is not homogeneous (Figure 4) [68].

Figure 4 shows that in the swollen state, when the alginate gels are immersed in pure water, the pores undergo opening to introduce this water into the interior of the composite. It is of note that swelling at the body temperature (37 ± 0.5 °C) produces a morphology with larger pores than swelling at 26 ± 0.5 °C (Figure 4) [68]. With the addition of more CNFs, higher tensile properties are achieved and high resistance when they are not in the swollen state [66]. The combination of zinc cations with GO presents itself as an exciting mixture for the medical industry and increases properties, such as thermal characteristics, degradation, and exact dielectric properties [104,232]. Single-walled carbon nanotubes (SWCNT) are used to promote breakage reinforcement and mechanical traction of many hydrogels [233,234]. Thus, alginate combined with SWCNTs also showed increased mechanical reinforcement, making these composites ideal candidates for many biomedical applications [235]. Carbon nanotubes (CNTs), in combination with alginate, can be used for drug portability due to greater flexural strength, more excellent stability, less drug leakage, and a much more sustainable release profile [236].

Multi-walled carbon nanotubes (MWCNTs) in alginate hydrogels increase porosity and decrease the degree of the degradability of this hydrogel [237]. However, CNFs have recently shown to be capable of accelerating the biodegradability of the hydrophobic poly(3-hydroxybutyrate-co-3-hydroxyvalerate) (PHBV) biopolymer [238].

CNTs, similar to other CBNS, reinforced the dehydration and swelling of alginate hydrogels [239]. The addition of GO into SA has increased structural, morphological, and thermal stability and improved mechanical and traction properties [240,241]. Moreover, 3D cross-linked networks of GO in the form of irregular tubes incorporated into alginate hydrogels showed mechanical improvements due to microstructural changes [69]. Reduced GO (rGO) increases properties just like normal GO or Gr, making it a good alternative material [242,243]. GO improves the compression performances of alginate hydrogels when applied in a small concentration (0.1% *w/w*) [4]. The physical properties of alginate can significantly change depending on the green synthesis followed [67]. Thus, novel routes to synthesize alginate using small concentrations of GO have been proposed to generate a measurable increase in water diffusion capacity and different mechanical traction characteristics, among other properties [67].

#### 3.1.4. Reinforcement with Nanoparticles

In addition to CBNs, a broad range of nanoparticles of different chemical nature, such as ceramic, metal/metal oxide, and polymeric nanoparticles can be dispersed in alginate matrix to form blends in the form of hydrogels, films, mats, fibers, or microcapsules with superior properties [244]. These composites with nanofillers have tailored functionality and promising physical, chemical, electrical, and biological properties than pristine ones. Thus, an enhancement strategy consisting of in situ reduction of silver nanoparticles (AgNPs) by sodium alginate to obtain a silver-loaded composite with enhanced mechanical and antimicrobial properties has been reported recently [245]. Alginate biofunctional films modified with melanin and zinc oxide/AgNPs showed enhanced tensile strength [246]. Alginate-based mats reinforced with ZnO nanoparticles were prepared via an electrospinning technique and subjected to a washing-cross-linking process composed of homogeneous nanofibers with a diameter of 100 ± 30 nm [247]. Nanocomposite films based on sodium alginate and polyaniline and TiO_2_ nanoceramic, synthesized by the solution casting method, showed promising mechanical, electrical, and antimicrobial activity for food packaging applications [248]. Preparation and characterization of polyaniline/sodium alginate-doped TiO_2_ nanoparticles showed promising mechanical and electrical properties and antimicrobial activity for food packaging applications [248]. ZnO and ZnO/CaO nanoparticles in alginate films were characterized mechanically to be used for food packaging [249]. Hydroxyapatite nanoparticles have also been used to enhance the mechanical performance and provide antibacterial properties to alginate films [250]. The use of titanium in combination with calcium alginate is a procedure that promotes immobilization efficiency, increasing the mechanical properties of the hydrogel up to three times compared to pure calcium alginate [251]. Montmorillonite nanoclay (MMT) and CaCl_2_-reinforced alginate-based nanocomposite film was prepared by the solvent casting method to increase internal mechanical and physicochemical properties [252,253]. The use of different nanoclays, such as MMT, laponite (LP), and sumecton (SUM), together with alginate, can be used as reinforcing agents for biomedical applications [74]. Biopolymers, such as alginate, are often combined with two types of nanoclays, such as kaolinite sheets and halloysite nanotubes, to enhance mechanical performance [254,255]. Incorporating nano-silica into an IPN produced with poly(acrylic acid) and alginate by UV polymerization increased its compressive strength and fracture resistance [63]. In bioprinting applications, cell-laden alginate–gelatin composite bio-ink with bioactive glass nanoparticles showed enhanced mechanical and biological properties [256].

### 3.2. Improvement of Thermal Properties

The thermal properties of alginate can be improved by incorporating nanomaterials with excellent thermal properties, such as CBNs or metallic nanoparticles, among others [257,258]. Thus, GO provides enhanced thermal resistance and stability to sodium alginate [240], and this enhancement increases with GO content [259]. Moreover, 3D cross-linked networks of GO showed enhanced thermal properties than single GO nanosheets when incorporated into calcium alginate composite hydrogels [69]. Furthermore, the thermal properties of calcium alginate/GO composites can be tailored following different chemical routes [67]. The introduction of other CBNs, such as rGO into calcium alginate, can efficiently improve its thermal stability [260]. Alginate-CuO nanocomposite showed enhanced thermal stability with respect to neat alginate. In addition, these composites exhibited antifungal activity [261]. Thermogravimetric analysis (TGA) showed that the thermal stability of alginate/AgNPs composite films increased distinctively compared with neat alginate films [262]. The addition of inorganic and organic nanofillers, such as MMT and cellulose nanocrystals, has improved the thermal capacity of the alginate [263,264]. The carboxymethyl konjac glucomannan polymer increased the physical properties, especially the thermal stability of SA hydrogels due to the intermolecular hydrogen bonds between both polymers [265]. The combination of SA, Gr, and polyvinyl alcohol (PVA) increased the thermal stability of the final composite [266,267]. Thermal stability can be improved by combining halloysite and alginate [268]. However, the union of alginate with glycerol decreased the thermal decomposition capacity of the final composite [269]. Zinc cross-linked alginate improved thermal stability compared to that of SA [270]. Molybdenum disulfide nanoleafs incorporated into alginate hydrogels can provide enhanced thermal resistance [271]. The thermomechanical properties of alginate films were improved by incorporating SiO_2_, PVA, and glycerol [272].

### 3.3. Enhancement of Electrical Properties

Many materials, such as Gr, CNTs, CNFs, AgNPs, polypyrrole (PPy), among others, are excellent electrical conductive materials [257,273,274]. Therefore, they can provide electrical conductivity to alginate when they are incorporated into its biopolymer matrix. However, in these composites, the percolation threshold; that is, the connectivity between the conductive nanomaterials incorporated into the biopolymer, plays a very important role [275]. Parameters, such as nanoparticle types and forms, synthesis methods, treatments, and dimensionality, as well as polymer types and dispersion methods, have impact on the percolation threshold and, thus, on the maximum conductivity of the composite. The development of conductive composites has great potential in biomedical applications that allow the application of electrostimulation [276,277]. In this context, various CNTs have been successfully applied to increase the electrical conductivity of alginate hydrogels [278]. Nanofibers composed of alginate and CNTs produced by electrospinning showed increased mechanical and electrical properties when a large amount of CNTs were added [279]. Oher graphene-based materials have been proposed to enhance the electrical properties of alginate [280,281,282,283,284,285]. Thus, very recently, rGO, which has an electrical conductivity close to that of graphene, has been used to be produce electroactive calcium–alginate/polycaprolactone/rGO nanohybrid hydrogels for skeletal muscle tissue engineering [208]. Alginate complexes with metals, such as Ba^2+^, Sr^2+^, Pb^2+^, Cd^2+^, or Zn^2+^, present electrical properties similar to semiconductor materials [286]. Other strategies followed to increase the electrical properties of alginate consisting of incorporating bentonite (BNT) clay into the alginate matrix to form SA/BNT composites [287]. These composites showed increased conductivity and dielectric constants with an increasing percentage of BNT in the SA matrix [287]. SA, PVA, and graphene nanospheres bonded by the electrospection technique provided improved electrical properties suitable for the fabrication of conductive scaffolds for nervous tissue engineering application [277]. Aligned and random fibers made of Gr, SA, and PVA have been proposed as conductive scaffolds for peripheral nerve engineering [276]. The results of this study revealed that the aligned fibrous scaffolds closely mimicked the anisotropic structure of the native sciatic nerve and electrical stimulation significantly enhanced PC12 cell proliferation. Another approach to increase the electrical properties of alginate consisted of adding PPy, by chemical polymerization [288]. This conductive polymer achieved an improvement of up to more than 10 times the electrical conductance compared to natural alginate.

### 3.4. Enhancement of Wettability

The wettability of a material surface is usually characterized by contact angle (CA) measurements between solid and liquid interfaces [67,289,290]. Hydrophilicity or wettability of biomaterials is considered a very important parameter for certain applications, such as cell adhesion in tissue engineering [291]. Alginate hydrogels have a high degree of hydrophilicity, so water droplets extend along the alginate film surface, with an average CA of less than 15° [292]. The incorporation of CBNs, such as GO nanosheets into calcium alginate, increases wettability [71]. The hydrophilicity of calcium alginate/GO composites can be tailored following different chemical routes [67]. Hydrophilic alginate-based multidentate biopolymers have been proposed for surface modification of CdS quantum dots [293]. A coating structure formed by alginate/Bioglass^®^ [294] and the addition of the water-soluble PVA polymer [295] showed suitable hydrophilic behavior for dental and orthopedic applications [296]. A study with alginate microcapsules showed that the smaller the size, the greater the wettability [297]. The addition of inulin increased wettability of microcapsules. Hydrophilic membranes consisting of an active alginate layer and supporting the chitosan layer on top of the base porous blended polyvinylidene fluoride (PVDF) membrane were prepared for pervaporation dehydration applications [298]. The blend of PVDF with 1% PMMA was shown useful in giving better surface properties for adhesion of the alginate and chitosan top layers. In addition, the high mechanical strength of PMMA [299] enhanced the mechanical performance of the alginate-based composite.

### 3.5. Enhancement of Water Sorption and Diffusion Properties

The enhancement of water sorption and diffusion properties is very desirable in certain fields, such as biomedical engineering and industrial bioprocesses that employ immobilized cells or enzymes in calcium alginate hydrogels (biocatalysts) [4]. The improvement of water diffusion in bioprocesses implies the enhancement of mass transport and, thus, the increase of industrial productivity. This enhancement is also very desirable in many biomedical applications, such as tissue engineering, which, due to mass transport, plays an important role in cell survival [300]. One of the strategies to improve water diffusion (up to more than six times) consists of adding a minuscule amount of GO (0.1% *w/w*) to calcium alginate, simultaneously improving its compression modulus by a multiple of four-fold [4]. It has been reported that the transport of water through graphene-based nanochannels is ultrafast [301,302]. However, the addition of GO produced a reduction in the swelling properties of the calcium alginate/GO hydrogel generated. The incorporation of low amounts of GO are desirable in order to reduce production costs as much as possible [4,71]. Significant improvement in liquid water diffusion is also achieved by adding CNFs to cross-linked calcium alginate [66]. However, the incorporation of CNFs also reduce the water sorption of calcium alginate. Swelling of the alginate hydrogel was considerably inhibited by the union of GO or CNF nanoparticles, the alginate polymer chains and divalent ions of Ca^2+^ [4,66].

Swelling biomaterials can considerably increase membrane permeability but have an adverse effect and significantly decrease membrane selectivity [303]. Water absorption by SA can be significantly reduced by introducing GO nanosheets [304,305]. This phenomenon is attributed to the formation of hydrogen bonds between oxygenated compounds, which produces improved resistance to swelling. In addition, the interaction between divalent metal ions, such as Ca^2+^ and oxygenated functional groups in the basal plane and edges of the GO nanosheets in the alginate composite, considerably reduces the water absorption capacity [67]. PVA biopolymer is added to alginate to reduce swelling because it is hydrophilic, compact, and has a high degree of crystallinity [303]. Therefore, a small amount of PVA improves swelling resistance and reduces the entry of water into the alginate structure. Swelling measurements can be used as indicators of the degree of crosslinking of alginate [306].

## 4. Enhancement of Biological Properties

Even though alginate possesses excellent biological performance, the enhancement of biological properties, such as biodegradation, antimicrobial activity, cell adhesion, proliferation, differentiation, and immunological challenges is very desirable in a wide variety of industrial fields.

### 4.1. Enhancement of Biodegradation

**Figure 5:**
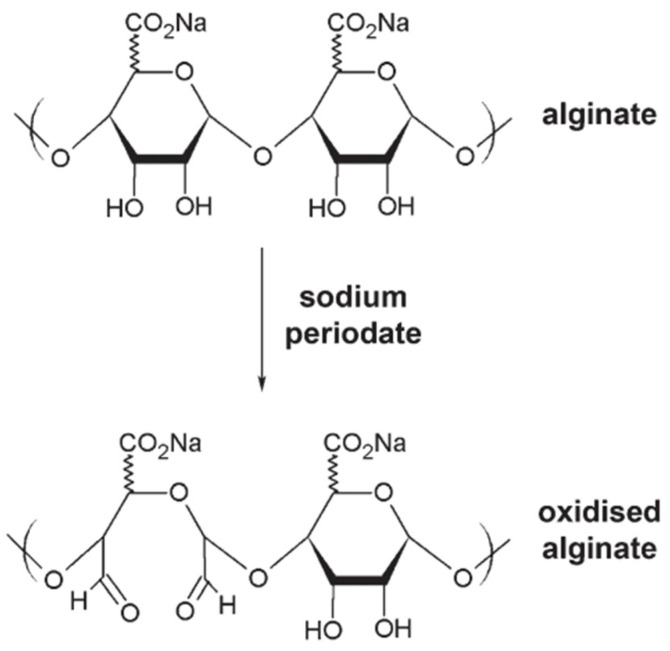
Sequential oxidation of alginate to yield alginate oxidized by sodium periodate [307].

The biodegradation of alginate can be enhanced because the biopolymer strands may be relatively oxidized after the addition of sodium periodate (Figure 5), breaking the bonds between the carbons of the cis-diol group and converting the chair conformation into an open chain. This reaction helps the degradation of the biopolymer backbone [307].

This oxidation, however, does not affect the alginate’s ability to produce alginate hydrogels by crosslinking with divalent ions, such as calcium [118]. The degradation behavior of the gel highly depends on the degree of oxidation, in addition to pH and media temperature.

**Figure 6:**
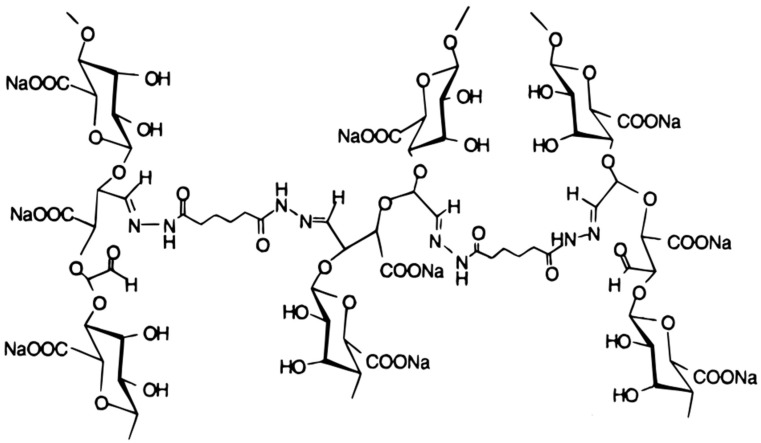
Poly(aldehyde guluronate) gels covalently cross-linked with adipic acid dihydrazide. Reprinted with permission from [308]. Copyright 2013 American Chemical Society.

Poly(aldehyde guluronate) (PAG) hydrogels can be prepared from alginate by acid hydrolysis and oxidation, followed by covalent cross-linking with adipic acid dihydrazide (AAD) (Figure 6) [308].

These hydrogels were degradable in aqueous media due to the hydrolysis of hydrazone bonds formed between the aldehyde of PAG and the hydrazide of AAD [308]. Furthermore, with the increase of the AAD crosslinker, a slow degradation process of the hydrogel is achieved. An alternative approach to control the degradation of alginate hydrogels consists of adjusting the molecular weight distribution [309]. Another strategy to regulate the degradation rates of hydrogels is by the modulation of the dissociation rates of the polymer chains via a size mismatch in the crosslinking zones [310]. The degradation of the alginate gel can also be regulated using a combination of partial oxidation of polymer chains and a bimodal molecular weight distribution of polymer [311]. The oxidation rate can modify many parameters of alginate hydrogels other than biodegradability, especially the photocrosslinked hydrogels formed by alginate oxidation and methacrylation (OMA) [112]. These OMAs can be used for tissue engineering and other biomedical applications because they are biodegrade and do not show any cytotoxic effects in human cells, such as human bone marrow-derived mesenchymal stem cells (hBMMSCs). Therefore, in the biomedical field, these enhancement strategies are critical. Thus, alginate oxidation increased the cell viability of corneal epithelial cells, which was even more improved with the additional introduction of type IV collagen [307]. When partially oxidized alginate is applied, it promotes the generation of structures that resemble cartilage, unlike gels that are not partially oxidized [118]. Another example is the more rapidly degrading oxidized binary hydrogels facilitating the formation of new bone tissues from transplanted bone marrow stromal cells, as compared with the non-oxidized hydrogels [309]. Encapsulation of fibroblasts has been shown to cause accelerated degradation of the alginate hydrogel [312].

### 4.2. Antimicrobial Activity

Alginate-based materials possess low or no toxicity and are capable of inactivating a wide variety of viruses affecting different organisms: in humans by the human immunodeficiency virus type 1, the hepatitis A, B, and C viruses, Sindbis virus, herpes simplex virus type 1 and 2, poliovirus type 1, rabies virus, rubella virus, and influenza virus; in mice by murine norovirus; in bacteria by the T4 coliphage, and in plants by the tobacco mosaic virus and the potato virus X [313]. Furthermore, biocompatible calcium alginate films, prepared by solvent casting and subsequent crosslinking with calcium cations, have recently shown antiviral activity against enveloped viruses, such as SARS-CoV-2 Delta variant [314]. The antiviral activity of these calcium alginate films is attributed to its compacted negative charges that may bind to viral envelopes inactivating membrane receptors. However, calcium alginate does not have antibacterial activity [71,133]. The antibacterial capacity of alginate-based materials is very desirable for certain biomedical applications, for example, to heal wounds, such as dressings, and for their introduction into the organism in the form of scaffolds for tissue engineering [315]. Combining alginate with materials with intrinsic antimicrobial properties such as zinc, silver, copper, or carbon nanomaterials, among others, constitutes an advanced strategy to achieve this goal.

#### 4.2.1. Zinc

The combination of zinc-based materials, such as ZnO nanoparticles with alginate, exhibited high antimicrobial capacities, of up to 99% efficiency in Gram-positive pathogens, such as *Staphylococcus aureus*, or 100% in Gram-negative bacteria, such as *Escherichia coli* [316]. This antibacterial capacity provides a tremendous advantage for food and clinical use [317]. Nano zinc oxide was impregnated effectively over cellulose fibers through sodium alginate matrix to produce next generation fibers with antibacterial activity [318]. Other preparations consisted of flexible and porous bandages made of alginate hydrogels with ZnO for healing wounds [319]. These bandages showed a directed biodegradable profile, antibacterial capacity, and rapid healing. Novel zinc alginate hydrogels prepared by internal setting method showed intrinsic antibacterial activity [320]. In addition, Zn can be used for alginate encapsulation to avoid infections [321]. However, it should be noted that the use of high concentrations of Zn^2+^ [104] or ZnO [319] produces remarkable cytotoxicity.

#### 4.2.2. Silver

Silver ions and silver nanoparticles are often used as an antimicrobial agents [322,323]. In fact, AgNPs are currently used in a broad range of industrial applications such as wound healing in biomedicine, food and textile industries, paints, household products, catheters, implants, cosmetics, and in combination with many types of materials to prevent infections [273]. 

Silver containing alginate shows antimicrobial activity, improves antioxidant capacity active, and reduces pro-inflammatory cytokine concentration [324,325]. In addition, the amount of free radicals and the use of this silver alginate in wound healing increase its effectiveness in infected wounds [326]. This hybrid material considerably reduced the growth of *S. aureus* [324]. Silver can be released from alginate into ions to kill bacteria [326]. A porous complex made of chitosan, alginate, and AgNP showed antimicrobial and anticancer properties [327]. However, extensive use of this metallic antimicrobial agent has produced bacterial resistance [328,329]. A compound formed by SA hydrogels and PVA and silver showed antibacterial activity [330]. Beads generated from a dual crosslinked PVA/SA/silver nanocomposite present a new structure that is cheap and exploits antimicrobial capability for food preservation [331]. AgNPs in sodium alginate and PVA increased mechanical capacity, and IC_50_ dose showed an increase in antibacterial and antifungal effects [332].

#### 4.2.3. Copper

Copper nanoparticles (CuNPs) incorporated into alginate provides antibacterial activity to neat alginate [333]. Therefore, recent studies have shown that the union of CuO with alginate increases structural properties and antibacterial activity, especially against *S. aureus* and *E. coli* [334]. The incorporation of copper-based materials, such as dendritic copper microparticles in the alginate biopolymer matrix, is low cost compared to other metals and ease of use [335]. Nanocomposites based on polypropylene non-woven fabric, alginate and copper oxides nanoparticles showed maximum reduction of tested microorganisms [336]. Other proposed antimicrobial platforms consisted of several combination of materials, such as bacterial cellulose/alginate/chitosan composites incorporating copper (II) sulfate [337], wool fabric treated with alginate/Cu^2+^ [31], polylactide/alginate/copper [338], alginate/copper systems on cotton and bamboo fabrics [339], or copper-doped bioglass/alginate [340]. In the field of additive manufacturing, porous materials made of copper–tungsten–silver alloys showed antiviral activity against a viral model of SARS-CoV-2 [341] and 3D-printed alginate/bacterial cellulose composite hydrogels with incorporated copper nanostructures showed antimicrobial capacity [342]. However, it is essential to be aware that several metals, such as Cu, have certain cytotoxicity, producing oxidative stress, so the concentration introduced into alginate hydrogels must be controlled [343].

#### 4.2.4. Carbon-Based Nanomaterials

**Figure 7:**
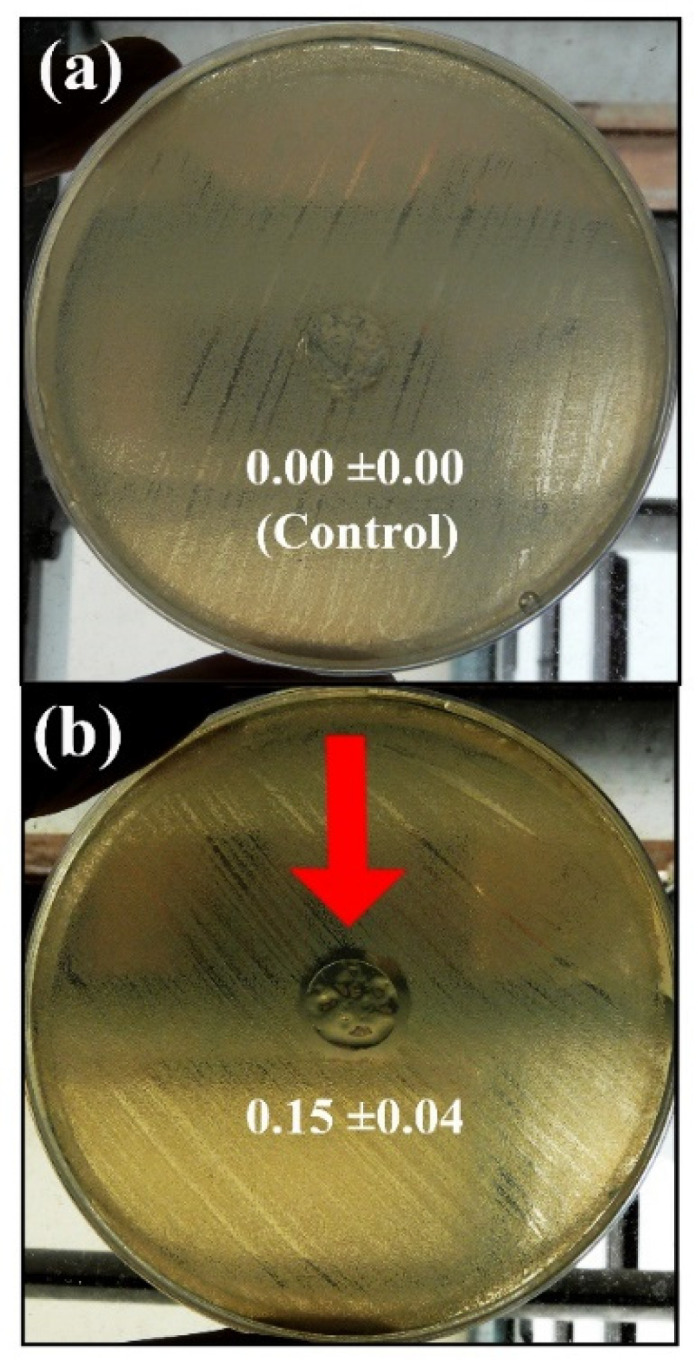
Results of the antibacterial test of CNFs in alginate films. Control alginate films without CNFs (**a**) and alginate films with a low amount (0.1% *w/w*) of CNFs (**b**) against the methicillin-resistant *Staphylococcus epidermidis* (MRSE) bacteria by the agar disk diffusion method at 37 °C after 24 h of incubation. The bacterial inhibitory halo produced by the antibacterial material film of calcium alginate/CNFs can be clearly observed (red arrow) [133].

Carbon-based nanomaterials are next generation materials that have recently been proposed to treat COVID-19 because they have shown antibacterial and antiviral activity against 13 enveloped positive-sense single-stranded RNA viruses, including SARS-CoV-2 [344]. They are broad-spectrum antimicrobial materials, capable of inducing tissue regeneration and characterized by a low risk of microbial resistance. Thus, the incorporation of a low amount (0.1% *w/w*) of a CBN, such as CNFs, into calcium alginate films showed antibacterial activity against the life-threatening methicillin-resistant *Staphylococcus epidermidis*, or MRSE (see antimicrobial inhibition zone in Figure 7b), and no cytotoxicity was observed in human keratinocyte HaCaT cells [133].

Other CNBs, such as GO (0.5% or 1% *w/w*) combined with alginate, exhibited high antibacterial properties against multidrug-resistant bacteria, such as MRSE, and other relevant pathogens, such as *S. aureus*, ensuring no cytotoxicity in human HaCaT cells [71].

CNFs incorporated into calcium alginate films enhanced its antiviral properties against bacteriophage T4 [345]. Furthermore, GO and CNFs combined with LED irradiation increased the antibacterial activity of these two nanomaterials [346]. Contrary to the above, incorporating GO at 1% concentration into zinc alginate films did not increase the bacteria-killing properties [104]. The antimicrobial activity of CNMs are attributed to different antimicrobial mechanisms, such as membrane stress, oxidative stress, entrapment, electron transfer, and photothermal hypotheses [347].

#### 4.2.5. Other Alternative Materials

Other alternative materials that can provide antimicrobial properties to alginate gels include nanoclays, quaternary ammonium compounds, lactoperoxidase systems, κ-carrageenan, chitosan, and cobalt (II), among others. Thus, alginate combined with two types of nanoclays leaves and halloysite nanotubes may promote the antimicrobial activity of the final complex [348,349]. Another antimicrobial method consists of complex binding of the alginate–quaternary ammonium complex by reaction of SA bound to (trimethoxysilyl)propyl-octadecyl dimethyl ammonium (TSA) with subsequent crosslinking with CaCl_2_ [350]. The lactoperoxidase system and cross-linked alginate hydrogels generate a characteristic antimicrobial activity [351]. Antimicrobial films have been developed based on alginate crosslinked with calcium and κ-carrageenan [352]. Antimicrobial chitosan-SA polyion complexes [353] and nanoparticles [354] have been developed. Alginate/chitosan particles with diclofenac by dropwise addition to the CaCl_2_ solution have been developed for biomedical applications [355]. Furthermore, a novel alginate derived cationic surfactant-cobalt(II) complex through the reaction of alginate with cationic surfactant showed good antimicrobial activity against Gram-positive and Gram-negative bacteria and fungi. Although, the antiviral properties of this complex have not been tested, in the field of additive manufacturing, porous materials made of cobalt-based superalloys showed potent antiviral activity against a viral model of SARS-CoV-2 [356].

### 4.3. Enhancement of Cell Adhesion, Proliferation, and Differentiation

Cell adhesion on alginate structures has been studied, and this characteristic is generally very low [68,135,357,358]. In this regard, several strategies have been developed to overcome this drawback of alginate gels for biomedical applications requiring cell adhesion, such as regenerative medicine and tissue engineering. Thus, peptide-coupled alginates obtained by chemical functionalization of alginates have shown to increase cell adhesion [359,360]. Sulfated alginates that resemble heparin have been developed for biomedical applications [361,362,363]. Heparin is a sulfated compound with a negative charge, formed via uronic acid dimers linked to glucosamine molecules through a bond that engages carbons 1,4 [364]. Heparin can mediate with different proteins and factors relevant to biological development [365]. Hydrogels presenting heparin are applied in an injectable form and can mediate different processes, such as those mentioned above, or deliver factors that aid cell growth in target tissues [365].

Cell adhesion and proliferation can also be increased with the incorporation of CNM into different biomaterials, such as PHBV [290,366]. Examples of these carbon nanomaterials are GO and CNFs, which, added in low concentrations, significantly increase cell adhesion and the proliferation capacity of the biomaterials. Other materials used in biomedicine that exhibited improved cell adhesion with the incorporation of GO are polycaprolactone [367] and gelatin [368]. However, alginate-based films with CNFs [68] or GO [135] showed non-cytotoxic effects in human HaCaT cells used, but did not significantly enhance cell adhesion, unlike the other biomaterials. Alginate supports do not produce an increase in cell adhesion capacity when non-hydrophilic CNFs or hydrophilic GO are introduced. However, the incorporation of these CBNs into alginate is capable of enhancing many other physicochemical and antibacterial properties [4,66,67,71,133]. Alginate-catechol is an adhesive gel capable of remaining adherent to endothelial cells under flow above physiological shear stress [369]. Furthermore, the alginate-catechol matrix exhibits enhanced mechanical stress strengthening and chemical stability that does not change its morphology even if the pH values vary over a large pH spectrum [370]. Recent studies have shown that enhancing properties, such as cell adhesion, cell spreading, and neurite outgrowth, are achieved by binding alginate with up to three laminin-active proteins [371].

Mouse embryonic stem cell culture using alginate hydrogels as 3D scaffolds efficiently supports neural differentiation [372]. However, enhancement strategies combining alginate with other biomaterials has shown successful results in this research area. Thus, human adipose-derived stem cells encapsulated in alginate/gelatin microspheres showed much higher cell proliferation as well as adipogenic differentiation compared with encapsulation in pure alginate [373]. Mineralized alginate matrices have also shown osteogenic differentiation of human mesenchymal stem cells [374].

Alginate–gelatin with mouse planta dermis bio-ink facilitates the proliferation, migration, and sweat gland cell differentiation of mouse mesenchymal stem cells (MSCs) [375]. That study demonstrated that the chemical constituents of a bio-ink play a critical role in promoting the migration and development of MSCs. Furthermore, the physical properties of a bio-ink and spatial conformation can promote the differentiation of MSCs into sweat gland cells [375]. Alginate–gelatin microcapsules have also shown to enhance bone differentiation of mesenchymal stem cells [376]. The combination of alginate with another natural hydrogel, agarose, improved the in vitro differentiation of human dental pulp stem cells in chondrocytes [377]. Therefore, further research must be performed to continue developing new advanced alginate-based materials with enhanced cell adhesion, proliferation, and differentiation.

### 4.4. Enhancement Immunoengineering Strategies

SA has potential application in enhancing immunity in biomedicine [378,379]. Although cell encapsulation in alginate gels is a very promising therapy for cell transplantation according to the research performed so far in this field, the few clinical trials based on cell encapsulation are still under evaluation [380]. Encapsulation of the transplanted cells can solve the problem of immune rejection, by providing a physical barrier between the transplanted cells and the recipient’s immune cells [381,382]. However, due to the difficulties encountered when trying to prevent the immune responses generated by the various microcapsule components, progress in the area has been slow [380]. In this regard, the immune responses produced by the alginate polymer can be minimized using ultrapure alginates [381]. A strategy consisting of incorporating fucoidan from *Fucus vesiculosus* in ultrapure alginate for microencapsulation of primary rat islets showed that both viability and glucose responsiveness of rat islets in these gel microcapsules were significantly higher compared to islets encapsulated in alginate alone [383]. Encapsulation and immunoengineering strategies combined with cell therapy have been applied as enhanced strategies to improve cell replacement therapies for management of type 1 diabetes (T1D) [381]. For example, a potential novel strategy to improve long-term survival of pancreatic islet grafts for T1D treatment consists of an immune regulatory 3D-printed alginate–pectin construct for immunoisolation of insulin producing β-cells [384]. The incorporation of immunomodulatory molecules in alginate capsules demonstrated to be for long-term engraftment and functioning of insulin-producing cells [385]. Thus, for example, the combination of alginate with crystalline GW2580, a colony-stimulating factor-1 receptor inhibitor, showed long-term release of the immunomodulator, which reduced fibrosis and facilitated glycemic control for xenogeneic islets transplantation in mice [386].

## 5. Porous Alginate Scaffolds for Tissue Engineering

**Figure 8:**
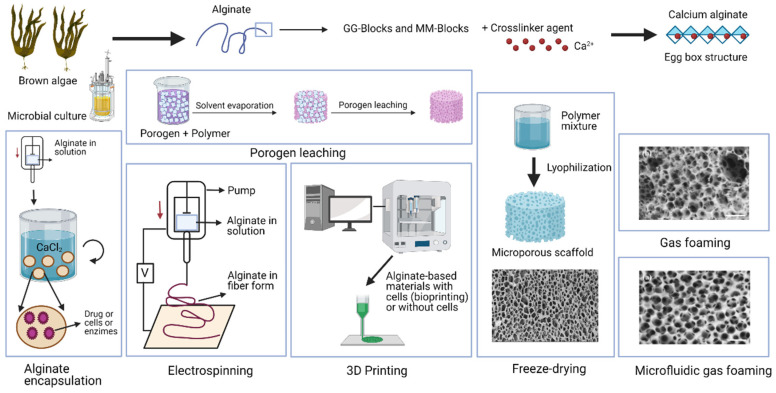
Summary illustration of extraction, crosslinking, central alginate manipulation, encapsulation, and scaffold formation techniques, such as electrospinning, 3D printing, freeze-drying, gas foaming, microfluidic gas foaming, and the porogen leaching technique. Created with BioRender.com.

The fabrication of polymer supports, usually called scaffolds, with a high degree of interconnected porosity, is required for tissue engineering applications [387,388,389,390,391,392,393,394]. Alginate scaffolds can be produced following several strategies, which include gas foaming and microfluidic gas foaming [395], electrospinning [396], the leaching technique [397], freeze-drying [398], and 3D printing of biomaterials with cells (bioprinting) or without cells [399], among other methods [400,401]. Figure 8 presents a summary illustration of alginate extraction from brown algae or microbial culture, crosslinking, and primary techniques for alginate manipulation, encapsulation, and scaffold fabrication methods.

The enhancement strategies developed so far to improve the physicochemical and biological properties of alginate are very important for the fabrication of next generation alginate scaffolds for tissue engineering applications. In this regard, for example, novel alginate scaffolds composed of porous alginate that incorporate tiny poly(lactic-co-glycolic acid) microspheres capable of controlling the release of angiogenic factors, such as a basic fibroblast growth factor, have been developed [402]. Alginate/hydroxyapatite (HAp) scaffolds have also been developed with 82% porosity to allow the growth of osteoblastic cell lines favoring a promising approach for bone tissue engineering applications [403].

The fabrication of alginate scaffolds by the electrospun technique is inexpensive and easy to process and build [404]. In this technique, it is possible to modify the sizes of the structures and the diameter of strands depending on the properties of the natural biopolymer alginate used [10]. Electrospinning of pure alginate is a well-known technique to produce alginate scaffolds with potential applications in tissue engineering [405]. However, enhancement strategies, such as the introduction of gelatin to reinforce alginate electrospun nanofibers, can be applied to achieve improved scaffolding and transplantation in corneal tissue engineering [406]. The structure formed by electrospun alginate/poly(ethylene) oxide (PEO) and Pluronic F127 as a surfactant generated a scaffold with marked porosity for tissue engineering [407]. The use of alginate has been proposed in conjunction with PEO and peptides to improve cell adhesion, which is essential for tissue engineering applications [408]. However, the problems begin when the alginate concentration increases because the structure becomes viscous and cannot be injected, and the solution needs to then introduce surfactants or cosolvents to mediate in the phase transition [404]. If only alginate-based materials are desired, the water-soluble PEO of the nanofibers formed can be eliminated by incubation in water [409]. Three-dimensional electrospun alginate can be produced in combination with another biodegradable polymer, poly (ε-caprolactone) (PCL). These composite scaffolds can be produced with determined pore sizes to allow enhanced viability and tissue regeneration [410]. Electrospun nanofibrous scaffolds reinforced with magnesium oxide nanoparticles showed improved physicochemical properties, such as resistance to traction and elasticity [411]. Improving the resistance and durability of alginate hydrogels by the application of a method based on the layer-by-layer electrospinning of nanofibers showed very promising results at a structural level for tissue engineering [228]. An innovative 3D nanofiber hydrogel composed of alginate bonded with polyaniline in nanofiber form to promote a more stable and reinforced structure for lithium-ion battery applications have been reported via in situ polymerizations instead of electrospinning [412].

The particle leaching method, or porogen technique, and the freeze-drying method were combined with calcium alginate beads and keratin was used to create a flexible structure for fibroblast proliferation [413]. This process is fast and easy, and porosity and size can be controlled to generate various scaffolds with these biopolymers [414]. Alginate-chitosan/hydroxyapatite polyelectrolyte complex porous scaffolds with mechanical resistance and thermal stability were developed by combining the formation of the polyelectrolyte complex (PEC) with freeze-drying [415]. A porous matrix, a scaffold of calcium alginate/gelatin with enhanced properties, was generated by combining porogen leaching and lyophilization, generating a microenvironment for cell adhesion, generation, and tissue regeneration [13]. The mixture of freeze-drying and leaching can increase the pore size of the final alginate scaffold [416]. Alginate can also be used in the form of microsphere porogens to produce porous scaffolds with another biopolymers, such as collagen [417]. Moreover, 3D printing technologies or additive manufacturing techniques are broadly used to fabricate scaffolds, such as fused deposition modeling (FDM) [418]. Thus, 3D printing was used to fabricate porous alginate/gelatin hydrogel scaffolds for tissue engineering [419]. Moreover, 3D printing can handle materials and cells, such as human chondrocytes with nanocellulose-alginate, while moving in the three axes allowing structures with volume for bioengineering applications [77]. Furthermore, 3D printing has been investigated as a promising technique to build tissues by applying microscopic structure control and macroscopic layer by layer production [420]. The 3D tracing technique is also applied to form cell-loaded porous alginate, and this technique is used to form scaffolds for bone problems or cartilaginous tissues [421]. PCL and alginate were combined to form a three-dimensional structure capable of withstanding cell activity and providing a mechanically stable material [422]. Alginate-based heart valves as scaffolding fabricated by the 3D bioprinting technique showed increased viability, correct dispersion, and cell retention [423].

Bioprinting or 3D bioprinting is a technique that allows the direct manufacturing of an artificial living tissue by combining biomaterials, cells, and growth factors using optical and different computational methods and NMR technology data of the tissue or organ to be copied [17]. The structural matrices are multicellular (bio-links) in a sequential layer-by-layer methodology based on these advanced technologies. Moreover, 3D printing can be used to obtain an automatic and reproducible production of living and functional 3D tissues that are more practical tools when carrying out drug experiments, toxicological studies, and even transplants [16]. However, not any material can be used for this purpose since it must meet specific biocompatibility requirements and basic structural and/or mechanical properties for which the most recommended are hydrogels such as alginate [424]. Thus, alginate-based hydrogels are the main biomaterials applied in generating 3D structures because they are polymer of natural origin, biodegradable, non-cytotoxic, and do not generate a response from the immune system, they are also economical compared to other biopolymers. Furthermore, this polymer is obtained from renewable sources, such as brown algae or microorganism culture [4]. Nevertheless, alginate also has its drawbacks. Thus, alginate degradation is slow and difficult to control [425]. This is a serious problem because, for a suitable tissue regeneration, the material must be degraded and allow the cells to generate extracellular components themselves that promote the surface matrix. In addition, the properties required for the manufacturing of different tissues are different, so it is usually necessary to combine alginate with other biomaterials to achieve optimal mechanical and structural properties in each case [424]. Thus, alginate combined with other biomaterials has a fundamental role in tissue regeneration, highlighting cartilage, bone [426], and vascular tissue [427]. Among the main peculiarities, it is still a challenge for the scientific community to reduce the “bench-to-bedside” gap for the proper functionality of bioprinted tissue. To this end, several research groups have been working in alginate constructions, such as alginate scaffolds with sustained release of the BMP-2 protein for osteogenicity by bioprinting [428], mesoporous bioglass/alginate scaffolds with porosity up to 70% [429], alginate scaffolds with calcium phosphate for osteochondral regeneration [430], and alginate scaffolds with CBNs such as GO [431].

Freeze-drying is an easy method to produce three-dimensional porous materials for regenerative purposes [432]. In-process freeze monitoring is essential for pore formation during scaffold production [433]. Many scaffolds are produced by this technique, such as alginate scaffolds with immobilized Arg-Gly-Asp (RGD) peptide [434,435], curcumin-loaded chitosan nanoparticles impregnated into collagen–alginate scaffolds [436], and alginate/poly (lactic-co-glycolic acid)/calcium phosphate cement scaffolds [437].

Sophisticated methods, such as the four-step process that consists of applying a preparation followed by crosslinking, and a step similar to freeze drying, freezing, and lyophilization, has been used to prepared chitosan–alginate as scaffolding material for cartilage tissue engineering [438]. Chitosan/alginate-based scaffolds have also been produced by thermally-induced phase separation and subsequent sublimation of the solvent [439], by in-situ co-precipitation containing different amounts (0, 10, and 30 wt.%) of HAp [440]. A coaxial structured collagen–alginate scaffolds was designed with an outer collagen and an inner alginate part [441]. These biocompatible scaffolds showed good structural stability and increased mechanical performance compared to pure collagen scaffold under a similar pore structure. Furthermore, they showed good cytotoxicity. Porous bony scaffolds coated with alginate–hydroxyapatite have been recently developed by a combination of several techniques that include coating, 3D printing and freeze-drying for femoral applications [442]. Alginate sulfate-based hydrogel/nanofiber composite scaffolds with controlled Kartogenin delivery have been proposed for tissue engineering [443]. Very recently, chitosan/alginate/hydroxyapatite hybrid scaffolds have been developed using 3D printing and impregnating techniques for potential cartilage regeneration [444]. Table 5 summarizes the most relevant alginate-based scaffolds showing the fabrication method, materials combined with alginate, pore size/shape, porosity, regenerative field, year of publication, and reference.

## 6. Conclusions and Future Perspectives

In this review, alginate biopolymer’s most relevant enhancement strategies, including the principles, chemical structure, gelation properties, chemical interactions, production, sterilization, purification, types, and alginate-based hydrogels developed so far, and scaffolds for regenerative medicine, have been exposed. Emphasis was placed on the materials and compounds combined with alginate to develop novel composite and nanocomposite materials with improved physicochemical and biological properties that solve the drawbacks of this well-known natural biomaterial. Alginate has been proposed as a natural antiviral material capable of inactivating enveloped viruses, such as SARS-CoV-2 for the current coronavirus pandemic. Therefore, the excellent properties and the increasing amount of enhancement strategies of this biopolymer render alginate-based materials a family with great potential in a broad range of fields, from the food industry to the most sophisticated biomedical technologies.

## Figures and Tables

**Table 1:** Application areas of alginate and its specific uses.

Area	Specific Use	Reference
Biotechnology, bioengineering, biomedicine, and clinical	Dressings for wounds and burns	[6,7]
	Heavy metal chelator	[8,9]
	Scaffolding in tissue engineering	[10,11,12,13]
	Controlled release	[14,15]
	3D bio-printing	[16,17]
	Prosthesis, dental molds and impression materials	[18,19,20,21]
	Immobilization of enzymes and cells	[2,22]
Pharmaceutical industry	Food supplements	[23]
	Treatment for gastric reflux	[24,25]
	Cancer therapy	[26,27]
Chemical, textile, packaging and construction industry	Cosmetics	[28,29]
	Textile inks	[30,31]
	Detergents	[32]
	Adhesives	[33,34]
	Welding	[35,36,37]
	Building insulation	[38]
	Biodegradable packaging	[39]
Food and drinks	Ice cream production	[40,41]
	Binder and thickener	[42,43]
	Beer foam stabilizer	[44,45]
	Confectionery and gastronomy in general	[46,47]
Aquaculture	Binder for food	[48,49]
Paper industry	Thickener	[39]
Arts and crafts	Taxidermy molds	[44,50]
Leisure industry	Protective masks	[51,52]

**Table 2:** The main alginate types from brown algae depend on the source, M/G ratio, molecular weight, and viscosity. Nd: not determined.

Source	M (%)	G (%)	Molecular Weight (kDa)	Viscosity (dL/g)	References
*Laminaria hyperborea*	25–35	75–65	91.902	6.4	[142,149,150]
*Laminaria digitata*	53–60	40–47	114–132	2.4	[90,113]
*Macrocystis pyrifera*	61	39	146–264	12.1	[142,151,152,153]
*Fucus vesiculosus*	53.4–59	41–46.6	125–154.9	2.5	[150,154,155]
*Sargassum fluitans*	54.2	45.8	300	6.3	[150,154,156]
*Sargassum vulgare*	44–56	44–56	110–194	5.26–9.10	[150,157,158]
*Laminaria japonica*	65–72	28–35	770	15.4	[44]
*Ascophyllum nodosum*	46	54	177.3	2.8	[142,150,155]
*Saccharina longicruris*	41	59	106.6	Nd	[142,155]
*Durvillaea antartica*	68–71	29–32	Nd	7.82	[136,142,159]

**Table 3:** Genes involved in the production of alginate by bacteria and their gene products.

Gene	Product	Reference
algA	Phosphomannose isomerase/GDP-mannose pyrophosphorylase	[167,168,169]
algB	ntrC	[170,171]
algC	Phosphomannomutase	[172]
algD	GDP-mannose dehydrogenase	[173]
algF	O-Acetylation	[174]
algG	Mannuronan C-5 epimerase	[174,175]
algI	O-Acetylation	[174]
algL	Alginate lyase	[176,177]
algR1	Regulatory molecules	[178]
algS	Anti σ factor	[179]

**Table 4:** Main alginate types produced from different bacterial cultures, M/G ratios, and characteristics of bacteria.

Source	M (%)	G (%)	Features	References
*Pseudomonas aeruginosa*	70	30	Mucoid biofilms	[184]
*Pseudomonas fluorescens*,	60–73	27–40	1.4–1.8 of polydispersity index	[185,186,187]
*Azotobacter vinelandii*	6–75	25–94	Encystment process or biofilm	[185,186]
*Pseudomonas putida*	78–63	22–37	1.5–1.8 of polydispersity index	[186,187]
*Pseudomonas mendocina*	74	26	Mucoid biofilms	[186,188]

**Table 5:** Alginate-based scaffolds: fabrication method, materials combined with alginate, pore size/shape, porosity, regenerative field, year, and reference.

Scaffold Fabrication Method	Materials Combined with Alginate	Pore Size/Shape	Porosity	Regenerative Field	Year	Ref.
Freeze-drying method	None	200–300 µm	90%	Tissue regeneration	2002	[433]
	Hydroxyapatite	150 µm	>82%	Bone	2004	[403]
	Chitosan	200 µm	84–88%	Cartilage	2008	[65]
	Sulfate	120 ± 30 µm	>90%	Vascularization	2009	[445]
	Poly (lactic-co-glycolic acid)/calcium phosphate	100–200 µm	89.24%	Bone	2009	[437]
	RGD	88 µm	>90%	Cartilage	2010	[434]
	RGD	50–100 µm	>90%	Cardiac tissue engineering	2011	[435]
	Curcumin, chitosan and collagen	50–250 µm	-	Diabetic wound healing	2016	[436]
	Collagen	200–700 µm	65–90%	Stem cell culture	2018	[417]
	PCL:gelatin electrospun mat, and kartogenin-PLGA nanoparticles	78.6 µm	92.4%	Tissue engineering	2021	[443]
3D printing/Bioprinting	MBG	300–420 µm	49–70%	Bone	2012	[429]
	PCL	388–499 µm	-	Bone	2012	[422]
	Calcium phosphate	200–900 µm	48–75%	Osteochondral regeneration	2013	[430]
	β-TCP	551–875 µm	23–52%	Bone tissue engineering	2014	[446]
	Tricalcium phosphate (TCP)	-	>80%	Bone	2016	[447]
	Gelatin	-	40–75%	Tissue regeneration	2016	[448]
	BFP1	-	-	Bone regeneration	2017	[449]
	Graphene oxide	-	-	Chondroinductive	2020	[431]
	Gelatin	<500 µm	60–70%	Bone regeneration	2021	[450]
	Polyethylene glycol	291.4 μm	-	Delivery of insulin	2022	[451]
Electrospinning	PEO	-	-	Tissue regeneration	2010	[408]
	Chitosan and PEO	-	-	Tissue regeneration	2011	[409]
	Gelatin	-	-	Corneal tissue engineering	2013	[406]
	PCL and ethanol treatment	-	-	Tissue regeneration	2013	[228]
	PCL	821 ± 55 µm	92%	Bone	2014	[410]
	Magnesium oxide	2–50 µm	Low	Tissue regeneration	2017	[411]
Porogen leaching	Poly(D, L-lactic acid)	450–900 µm	84.24–90.75%	Bone	2008	[452]
	Gelatin	204 ± 58 µm	97.26 ± 0.18%	Cell culture for regeneration	2015	[13]
	Collagen	700 µm	-	Cell cultures	2018	[417]
	Gelatin/PVA	104.5 ± 15.9 µm	74.5 ± 15.9%	Meniscus fibrocartilage	2018	[453]
	Vaterite/Crystals	10–500 µm	-	Tissue regeneration	2019	[454]
Four-step process: preparation, cross-linking, freezing and lyophilization	-	50–200 µm	>90%	Vascularization and generation of embryos	2004	[455]
	Chitosan	100–300 µm	-	Cartilage	2005	[438]
Solution and crosslinking	Fibroblast growth factor	100–500 µm	>90%	Vascularization	2003	[402]
Thermally induced phase separation and subsequent sublimation of the solvent	Chitosan	100–300 µm	91.94 ± 0.9%	Bone	2005	[439]
Co-precipitation	HAp/chitosan	50–100 µm	79–85%	Bone and other tissues	2008	[440]
Sol–gel synthesis Surfactant foaming	Bioactive glass/polyvinyl alcohol	200–500 µm	-	Trabecular bone	2009	[456]
Homogenizing interpolyelectrolyte complex method	Chitosan on PEC gel	100 µm	-	Release of growth factor for tissue regeneration	2009	[457]
Lyophilization	Chitosan/Hydroxyapatite	80–200 µm	>70%	Tissue regeneration	2010	[415]
Core/shell nozzle of a cryogenic co-extrusion process	Collagen	100–200 µm	>90%	Skin tissue regeneration	2011	[441]
Modified Solid-Freeform	Cells (MC3T3-E1)	300 µm	-	Tissue regeneration in general	2012	[458]
Three monitorized precision linear stages	Chitosan	-	66%	-	2014	[459]
Binary polymer system	Felodipine Fibroin	-	49–62%	Silk fibroin	2020	[460]
Solvent casting technique	TiO_2_/Chitosan	None	-	Bone regeneration	2020	[461]
3D Printing (FDM)/freeze-drying/coating	PLA and hydroxyapatite	Circle	44–36%	Bone regeneration	2021	[442]
3D printing and impregnating techniques	Chitosan/alginate/hydroxyapatite	2–3 mm	-	Cartilage regeneration	2022	[444]

## Data Availability

Not applicable.
